# Ginsenoside Rg3 Attenuates Lipopolysaccharide-Induced Acute Lung Injury via MerTK-Dependent Activation of the PI3K/AKT/mTOR Pathway

**DOI:** 10.3389/fphar.2018.00850

**Published:** 2018-08-02

**Authors:** Jing Yang, Senyang Li, Luyao Wang, Fen Du, Xiaoliu Zhou, Qiqi Song, Junlong Zhao, Rui Fang

**Affiliations:** ^1^State Key Laboratory of Agricultural Microbiology, College of Veterinary Medicine, Huazhong Agricultural University, Wuhan, China; ^2^Hubei Center for Animal Diseases Control and Prevention, Wuhan, China; ^3^College of Animal Science and Veterinary Medicine, Tianjin Agricultural University, Tianjin, China

**Keywords:** acute lung injury, lipopolysaccharide, ginsenoside Rg3, inflammatory, MerTK

## Abstract

Acute lung injury (ALI) is a common clinical disease with high morbidity in both humans and animals. Ginsenoside Rg3, a type of traditional Chinese medicine extracted from ginseng, is widely used to cure many inflammation-related diseases. However, the specific molecular mechanism of the effects of ginsenoside Rg3 on inflammation has rarely been reported. Thus, we established a mouse model of lipopolysaccharide (LPS)-induced ALI to investigate the immune protective effects of ginsenoside Rg3 and explore its molecular mechanism. In wild type (WT) mice, we found that ginsenoside Rg3 treatment significantly mitigated pathological damages and reduced myeloperoxidase (MPO) activity as well as the production of pro-inflammatory cytokines tumor necrosis factor-α (TNF-α), interleukin-1β (IL-1β) and interleukin-6 (IL-6); furthermore, the production of anti-inflammatory mediators interleukin-10 (IL-10) and transforming growth factor-β (TGF-β), polarization of M2 macrophages and expression levels of the phosphorylation of phosphatidylinositol 3-hydroxy kinase (PI3K), protein kinase B (PKB, also known as AKT), mammalian target of rapamycin (mTOR) and Mer receptor tyrosine kinase (MerTK) were promoted. However, there were no significant differences with regards to the pathological damage, MPO levels, inflammatory cytokine levels, and protein expression levels of the phosphorylation of PI3K, AKT and mTOR between the LPS treatment group and ginsenoside Rg3 group in MerTK^-/-^ mice. Taken together, the present study demonstrated that ginsenoside Rg3 could attenuate LPS-induced ALI by decreasing the levels of pro-inflammatory mediators and increasing the production of anti-inflammatory cytokines. These processes were mediated through MerTK-dependent activation of its downstream the PI3K/AKT/mTOR pathway. These findings identified a new site of the specific anti-inflammatory mechanism of ginsenoside Rg3.

## Introduction

Acute lung injury (ALI), is a common clinical disease with a high morbidity rate in both humans and animals, which causes injury to the alveolar epithelial cells and pulmonary capillary endothelial cells because of non-cardiogenic factors, and it can further develop into its severe stage, acute respiratory distress syndrome (ARDS) (Ferguson et al., 2005; Niu et al., 2015; Chen et al., 2017). The morbidity rate of ALI is 38.5%, and the high morbidity rate of ARDS even reaches 41.1% (Rubenfeld et al., 2005). Therefore, it is an urgent need to find more effective medicines to cure ALI, reducing the high morbidity. Lipopolysaccharides (LPSs) are considered to have an important impact on the inflammatory response of ALI and widely used to establish ALI models (Zhu et al., 2017).

In the inflammatory response induced by LPSs, neutrophils and macrophages are recruited, which can eliminate pathogens and produce soluble mediators (Geissmann et al., 2010). Macrophages can be polarized under the stimulation of different microenvironments, and modify their functions between M1 macrophages, which can produce inflammatory mediators and chemokines, and M2 macrophages, which can mediate anti-inflammatory mediators and tissue injury repair (Martinez et al., 2009; Mills, 2012; Sorgi et al., 2017). Mer receptor tyrosine kinase (MerTK) belongs to a member of the Tyro3-Axl-MerTK (TAM) receptor family which shares a common ligand growth arrest-specific protein 6 (Gas6) (Lee et al., 2012b). MerTK is obviously expressed on monocytes, as well as, epithelial and reproductive tissues, and it plays an important role in regulating immune functions such as suppressing the innate immune response and mediating the clearance (efferocytosis) of apoptotic cells (Triantafyllou et al., 2017). It has been reported MerTK that has an inhibitory effect on LPS-induced ALI (Lee et al., 2012a). The phosphatidylinositol 3-hydroxy kinase/protein kinase B/mammalian target of rapamycin (PI3K/AKT/mTOR) pathway plays an important role in cell growth, proliferation, apoptosis and autophagy as a crucial intracellular signal transduction pathway (Zhang et al., 2016). It has been demonstrated that the inflammatory response induced by LPS was suppressed by the PI3K/AKT/mTOR pathway (Tsukamoto et al., 2008).

**FIGURE 1:**
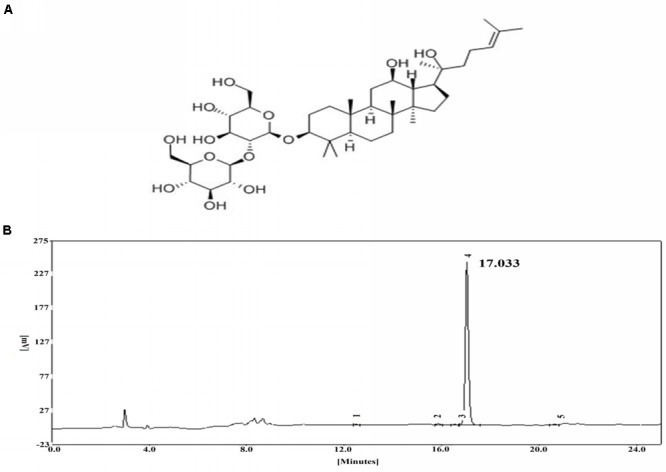
**(A)** Chemical structure of ginsenoside Rg3. **(B)** HPLC chromatogram of ginsenoside Rg3.

Ginsenoside Rg3 (**Figure 1A**) is a type of steroid compound that is extracted from ginseng, and it is supposed to have anti-tumor, anti-inflammatory and anti-fatigue activities (Schmidt et al., 2000; Shirasawa et al., 2004). The activity of ginsenoside Rg3 is closely related to pro-inflammatory cytokines [tumor necrosis factor-α (TNF-α), interleukin-1β (IL-1β) and interleukin-6 (IL-6)], cyclooxygenase-2 (COX-2) and the NF-κB pathway that plays an important role in inflammation (Ethridge et al., 2002). Some studies have reported that ginsenoside Rg3 has an immune regulatory effect on LPS-induced ALI (Kim et al., 2013), while little is known about the specific mechanism involved, and whether MerTK takes effect during the inflammatory response is not clear. The present study aims to make it clear whether MerTK takes part in the immune regulatory effect of ginsenoside Rg3 in ALI induced by LPS and illuminate the potential mechanism.

## Materials and Methods

### High-Performance Liquid Chromatography (HPLC)

High-performance liquid chromatography was employed to assess the purity of ginsenoside Rg3 by using an EChrom2000 DAD data system (Elite, Dalian, China) (**Figure 1B**).

### Animals and Groups Treatments

Sixty 6- to 8-week-old male wild type (WT) C57Bl/6 mice (weighing 20–25 g) and sixty male MerTK^-/-^ C57Bl/6 mice (weighing 20–25 g) were used in the study and were purchased from the Hubei Provincial Center for Disease Control and Prevention (Hubei, China). All of the mice were fed food and water under specific pathogen-free conditions at approximately 24 ± 1°C with a 40–80% relative humidity. All experiments were carried out in accordance with guidelines from the Laboratory Animal Research Center of Hubei province. This protocol was approved by the Ethical Committee on Animal Research at Huazhong Agricultural University (HZAUMO-2015-12). All efforts were made to minimize the suffering of animals as much as possible.

Ginsenoside Rg3 (purity ≥ 98%) was obtained from the Yuanye Biotechnology Company (Shanghai, China). LPS (*Escherichia coli* 055:B5) was purchased from Sigma (St. Louis, MO, United States). Dexamethasone (DEX) was purchased from Biosharp (Wuhan, China). The method of establishing the LPS-induced ALI model was performed as described previously (Hu et al., 2017). Briefly, 10 μg of LPS in 50 μL of sterile phosphate buffered saline (PBS) was administered intranasally into the nose to induce ALI. Ginsenoside Rg3 and DEX were intraperitoneally injected 1 h before LPS treatment. These mice were randomly divided into 8 groups as follows.

(A_1_) Control group (CG): The WT mice were treated with 50 μL of PBS.
(A_2_) DEX group (DEX): 1 h before LPS treatment, the WT mouse model of ALI was intraperitoneally injected with DEX at 5 mg/kg.
(A_3_) Ginsenoside Rg3 group (GG): 1 h before LPS treatment, the WT mouse model of ALI were intraperitoneally injected with ginsenoside Rg3 at 30, 20, and 10 mg/kg.
(A_4_) LPS group (LPS): The WT mouse model of ALI without drug treatment.
(B_1_) Control group (CG): The MerTK^-/-^ mice were treated with 50 μL of PBS.
(B_2_) DEX group (DEX): 1 h before LPS treatment, the MerTK^-/-^ mouse model of ALI was intraperitoneally injected with DEX at 5 mg/kg.
(B_3_) Ginsenoside Rg3 group (GG): 1 h before LPS treatment, the MerTK^-/-^ mouse model of ALI was intraperitoneally injected with ginsenoside Rg3 at 30, 20, and 10 mg/kg.
(B_4_) LPS group (LPS): The MerTK^-/-^ mouse model of ALI without drug treatment.

After treatment, all of the mice were euthanized by inhalation of the CO_2_. The lung tissues were collected 12 h after LPS induction, and stored at -80°C until analysis.

### Histopathological Evaluation

Lung tissues were acquired and fixed with 4% paraformaldehyde for 2 days. Subsequently, individual lobes of tissues biopsy material were placed in processing cassettes, dehydrated in a serial alcohol gradient, embedded in paraffin, and sectioned at a thickness of 4 μm. The 4-μm-thick lung tissue sections were dewaxed in xylene, rehydrated through decreasing concentrations of ethanol, and washed in PBS. Then the lung tissue sections were stained with hematoxylin and eosin (H&E), the cell nucleus were stained with hematoxylin and the cytoplasm was stained with eosin. After staining, sections were dehydrated through increasing concentrations of ethanol and xylene, finally the sections were sealed by neutral balsam for further analysis. The histopathological changes were observed with a light microscopy (Olympus, Japan).

### Immunofluorescence

The paraffin sections were repaired with EDTA Buffer and washed with PBS three times. Then the paraffin sections were incubated in 3% hydrogen peroxide solution at room temperature in the dark for 10 min to eliminate endogenous peroxidases, and blocked in 5% BSA for 20 min. The paraffin sections were incubated at 4°C overnight with 50 μL of primary antibodies against p-MerTK (1:150 dilutions, Bioss, United States). Subsequently, the paraffin sections were incubated with the secondary antibodies (1:50 dilutions, Aspen, China) at 37°C for 50 min. After staining, fluorescence microscope coupled with the MicroPublisher imaging software platform (Q-imaging) was used to observe the fluorescence intensity.

### MPO Assay

The lung tissues were harvested 12 h after LPS stimulated, and the tissues were homogenized with reaction buffer for myeloperoxidase (MPO) levels assay using an MPO commercial sandwich enzyme-linked immunosorbent assay (ELISA) kit (Eton Bioscience, San Diego, CA, United States) according to the manufacturer’s instructions. The detailed method is as follows: 100 μL of MPO standard solutions and 100 μL of samples were added to the proper wells, and then, they were incubated at 37°C for 90 min. One hundred microliters of biotinylated anti-mouse MPO antibody working solution was added to each well, and the plate was incubated at 37°C for 60 min. Each well was then washed with 100 μL of PBS 3 times. Subsequently, avidin-biotin-peroxidase complex working solution was added to each well and the plate was incubated at 37°C for another 30 min. Finally, 90 μL of TMB color developing agent and 100 μL of TMB stop solution were added to each well, and the absorbance of each well was measured at 450 nm using a microplate reader (Bio-Rad Instruments, Hercules, CA, United States).

### Cell Culture and Treatment

RAW264.7 cells were acquired from the American Type Culture Collection (ATCC TIB-71^TM^). The cells were cultured in DMEM supplemented with 10% FBS and were incubated continually at 37°C with 5% CO_2_. Ginsenoside Rg3 was dissolved in dimethyl sulfoxide (DMSO), and then diluted with DMEM to different concentrations for cell experiments. The final concentration of DMSO was less than 0.1% v/v. The cells were treated in groups as follows: (1) CG: cells without any treatment; (2) DEX, cells were incubated with DEX (100 μg/ml) as the positive control; (3) GG, cells were incubated with 100, 50, or 25 μg/ml ginsenoside Rg3 for 1 h, and then, the cells were stimulated by 2 μg/ml LPS for 12 h, and (4) LPS-stimulated group, cells were only stimulated with 2 μg/ml LPS.

### Cell Viability Assay

The cell viability was evaluated with using the MTT assay. RAW264.7 cells were seeded in 96-well plates for 12 h and then incubated with or without ginsenoside Rg3 (25, 50, and 100 μg/mL) or DEX (100 μg/mL). After 12 h of incubation, 20 μL of MTT was added into each well. Subsequently, the medium was removed, and 150 μL of DMSO was added into 96-well plates, which were agitated on the shaker table for 10min. The optical density (*OD*) value was determined at 490 nm using a microplate reader.

### ELISA Assay

The effect of ginsenoside Rg3 on the expression levels of pro-inflammatory cytokines and anti-inflammatory cytokines induced by LPS were measured using mouse TNF-α, IL-1β and IL-6, interleukin-10 (IL-10) and transforming growth factor-β (TGF-β) ELISA kits (BioLegend, United States) according to the manufacturer’s protocols. Fifty microliters of a serial dilution gradient standard or sample was added to the appropriate wells and incubated at room temperature for 2 h. Then, the plate was washed 4 times with 1 × wash buffer. Subsequently, 100 μL of detection antibody solution was added to each well and incubated at room temperature for 1 h. After washing the plate 4 times with 1 × wash buffer, 100 μL of avidin-HRP solution was added to each well and incubated at room temperature for 30 min. One hundred microliters of substrate solution and 100 μL of stop solution were added to each well in turn; finally, the *OD* value was determined at 450 nm using a microplate reader.

### Quantitative Real-Time Polymerase Chain Reaction (qRT-PCR) Assay

The total RNA was extracted from RAW264.7 cells using Trizol reagent (Transgen Biotech, China) following the manufacturer’s protocols. Then, the total RNA (1 μg) was reverse-transcribed to cDNA using the PrimeScript RT Reagent Kit with gDNA Eraser (TaKaRa, Japan). Gene-specific primers for quantitative real-time PCR were designed using Premier 7.0 software (Premier Biosoft International, Palo Alto, CA, United States), and the primers used are presented in the **Table 1**. Quantitative real-time PCR was performed on an ABI Stepone Plus real-time PCR instrument using SYBR Green qPCR Master Mix (TaKaRa, Japan). The thermal profile was as follows: 5 min at 95°C, 40 cycles of 95°C for 30 s, 59°C for 15 s, and 72°C for 20 s. β-actin was used to normalize all real-time PCR data, and the blank control was set to 1. The relative gene expression was calculated using the 2^-ΔΔct^ computing method.

**Table 1:** Primers used for qRT-PCR.

Name	Primer sequence (5′-3′)	GenBank	Product
		accession	size
		number	(bp)
TNF-α	CCCTCACACTCAGATCATCTTCT	NM_000594.3	61
	GCTACGACGTGGGCTACAG		
IL-1β	CCTGGGCTGTCCTGATGAGAG	NM_008361.4	131
	TCCACGGGAAAGACACAGGTA		
IL-6	GGCGGATCGGATGTTGTGAT	NM_031168.1	199
	GGACCCCAGACAATCGGTTG		
IL-10	TTGAATTCCCTGGGTGAGAAG	NM_000572.2	95
	TCCACTGCCTTGCTCTTATTT		
TGF-β	CTGGATACCAACTACTGCTTCAG	NM_000660.6	198
	TTGGTTGTAGAGGGCAAGGACCT		
β-actin	ACGGCCAGGTCATCACTATTG	NM_001101.4	71
	CAAGAAGGAAGGCTGGAAA		

### Flow Cytometry

The RAW264.7 cells were harvested from a 6-well plate and washed with cold PBS three times. Then, the cells were stained with PE Rat Anti-Mouse F4/80 (565410, BD, United States), FITC Rat Anti-Mouse CD11b (557396, BD, United States), APC-Cy7 Hamster Anti-Mouse CD11c (561241, BD, United States), PE-Cy7 Rat Anti-Mouse CD16/CD32 (560829, BD, United States) and Rat Anti-Mouse CD206 (565250, BD, United States) antibodies at 4°C for 30 min. After staining, the cells were washed with PBS three times and resuspended with 100 μL of PBS for FACS analysis using a BD FACSAria II instrument (BD, United States). The data were further analyzed using Flowjo software 7.6.

### Western Blot Analysis

The total protein was extracted from lung tissues and RAW264.7 cells using RIPA reagent (Beyotime Biotechnology, China) following the manufacturer’s protocols. The protein concentrations were determined using the BCA protein assay kit (Beyotime Biotechnology, China). Equal amounts (50 μg) of proteins were separated with 10% SDS-PAGE. Then, the proteins were transferred to polyvinylidene difluoride (PVDF) membranes, and the PVDF membranes were blocked in 2% BSA in TBST at room temperature for 2 h. The membranes were incubated at 4°C overnight with primary antibodies against PI3K, AKT, mTOR, p-PI3K, p-AKT, p-mTOR, β-actin (1:1000 dilutions, CST, United States), MerTK and p-MerTK (1:1000 dilutions, Bioss, United States), with β-actin used as a control. Subsequently, the membranes were washed with TBST five times and incubated with the secondary antibodies (1:1000 dilutions, Beyotime Biotechnology, China) at room temperature for 1 h. Finally, the protein levels were detected with an ECL Plus Western Blotting Detection System (Image Quant LAS 4000 mini, United States).

### Statistical Analysis

All the values were presented as the mean ± SEM. One-way ANOVA followed by Dunnet’s *post hoc* test was used for analyzing the differences between groups. A value of *P* < 0.05 was defined as statistically significant.

## Results

### Anti-inflammatory Effects of Ginsenoside Rg3 in WT Groups

**FIGURE 2:**
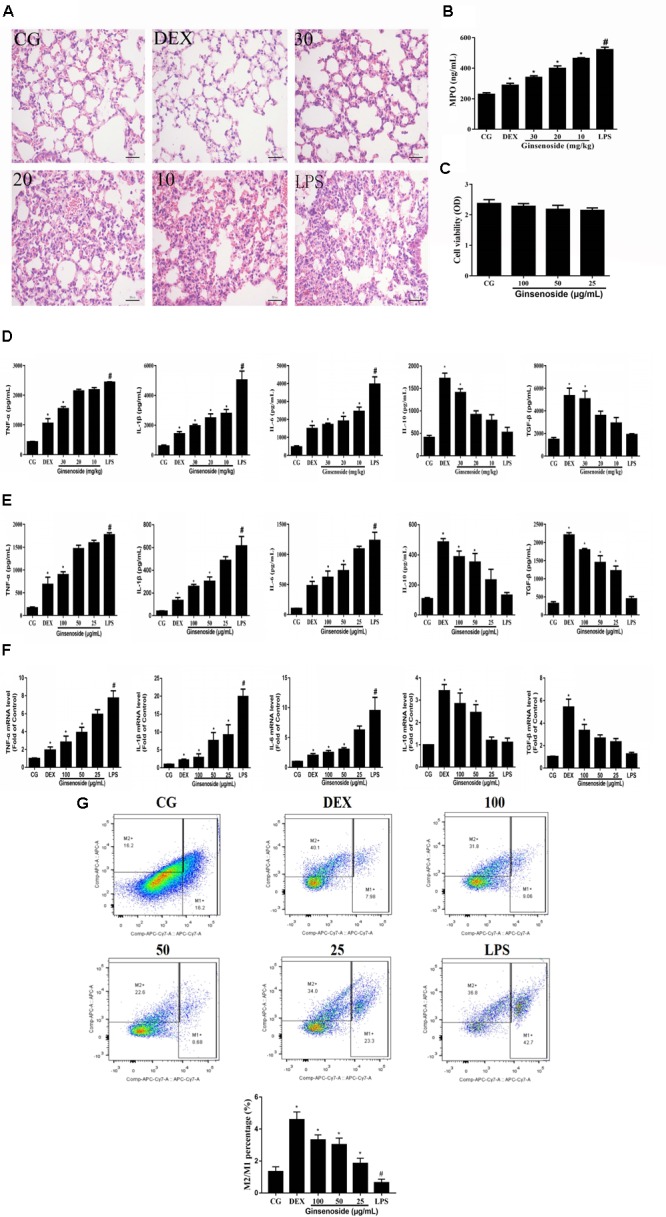
Anti-inflammatory effects of ginsenoside Rg3 in WT mice. **(A)** Histopathological analysis in lung tissues in WT mice. **(B)** The production of MPO of lung tissues in WT mice. **(C)** Effects of ginsenoside Rg3 on cell viability. **(D)** The proteins expression levels of TNF-α, IL-1β, IL-6, IL-10 and TGF-β in lung tissues were measured by ELISA. **(E)** The proteins expression levels of TNF-α, IL-1β, IL-6, IL-10, and TGF-β in RAW264.7 cells were measured by ELISA. **(F)** The mRNA expression levels of TNF-α, IL-1β, IL-6, IL-10, and TGF-β in RAW264.7 cells were measured by qPCR. β-actin was used as a control. **(G)** Effects of ginsenoside Rg3 on LPS-induced macrophage polarization in RAW264.7 cells. M1 macrophages were labeled with FITC Rat Anti-Mouse CD11b, PE Rat Anti-Mouse F4/80, APC-Cy7 Hamster Anti-Mouse CD11c and PE-Cy7 Rat Anti-Mouse CD16/CD32 antibodies. M2 macrophages were labeled with PE Rat Anti-Mouse F4/80, FITC Rat Anti-Mouse CD11b and Rat Anti-Mouse CD206 antibodies. The M2/M1 ratio in RAW264.7 cells was analyzed by flow cytometry. Gating was performed on F4/80^+^ cells. CG is the control group. LPS is the LPS-stimulated group. DEX is the dexamethasone group. Ginsenoside (10, 20, and 30) represent ginsenoside Rg3 (10, 20, and 30 mg/kg) + LPS in animals and Ginsenoside (25, 50, and 100) represent ginsenoside Rg3 (25, 50, and 100 μg/mL) + LPS in cells, respectively. The data are presented as the mean ± SEM of three independent experiments. ANOVA, *p* < 0.01, *post hoc* #*p* < 0.05 vs. CG. *^∗^p* < 0.05 vs. LPS group.

Lung tissues were harvested 12 h after LPS induction and then fixed for H&E staining, the results are shown in **Figure 2A**. In WT mice groups, there were no histological changes in the control group, while the group induced by LPS showed significant histological changes characterized by alveolar structure destruction, hemorrhage and inflammatory cell infiltration. The ginsenoside Rg3 treatment group significantly improved lung injury, and the DEX treatment group similarly showed significantly less lung injury.

The production of MPO was measured to assess the neutrophil infiltration in the lung tissues in the present study. As is shown in **Figure 2B**, MPO levels significantly increased in the LPS-stimulated group when compared with the control group. The increases of MPO levels were significantly suppressed by ginsenoside Rg3.

To assess the cytotoxicity of ginsenoside Rg3 on RAW264.7 cells, the MTT assay was used to measure the cell viability. The results demonstrated that the cell viability was not affected by ginsenoside Rg3 (**Figure 2C**). The effects of ginsenoside Rg3 on the production of cytokines in lung tissues and RAW264.7 cells were measured by an ELISA assay. The results in lung tissues showed that the expression levels of TNF-α, IL-1β and IL-6 were significantly increased after LPS treatment, while the levels of IL-10 and TGF-β were significantly decreased. In contrast, the increases of pro-inflammatory cytokines (TNF-α, IL-1β and IL-6) were obviously inhibited, and the decreases of anti-inflammatory cytokines (IL-10 and TGF-β) were significantly promoted by ginsenoside Rg3 and DEX (**Figure 2D**). The same results were observed in the RAW264.7 cell groups (**Figure 2E**). Likewise, the results of RAW264.7 cells measured by the qRT-PCR assay were consistent with those of the ELISA assays. The mRNA expression levels of TNF-α, IL-1β and IL-6 were significantly increased, and the mRNA expression levels of anti-inflammatory cytokines IL-10 and TGF-β were decreased after LPS stimulation compared with the control group. In contrast, ginsenoside Rg3 treatment suppressed the increases of pro-inflammatory cytokines (TNF-α, IL-1β and IL-6) as well as the decreases of anti-inflammatory cytokines (IL-10 and TGF-β) in a dose-dependent manner (**Figure 2F**).

As an important class of immune cells, murine macrophage RAW264.7 cells, have been widely employed to mimic the inflammatory response to evaluate the curative effect of an anti-inflammatory drug in LPS-induced ALI *in vitro* (Liu et al., 2017; Yang et al., 2017). Thus, we employed RAW264.7 cells to investigate the polarization of activated macrophages. From the results, it could be seen that the ratio of M2/M1 was obviously decreased after LPS treatment compared with the control group. However, in ginsenoside Rg3 treatment groups, the M2/M1 ratios were remarkably increased despite LPS stimulation. These results revealed that ginsenoside Rg3 treatment may exert an anti-inflammatory effect by promoting M2 polarization (**Figure 2G**).

### Effects of Ginsenoside Rg3 Treatment on LPS-Induced Activation of the PI3K/AKT/mTOR Pathway in WT Groups

**FIGURE 3:**
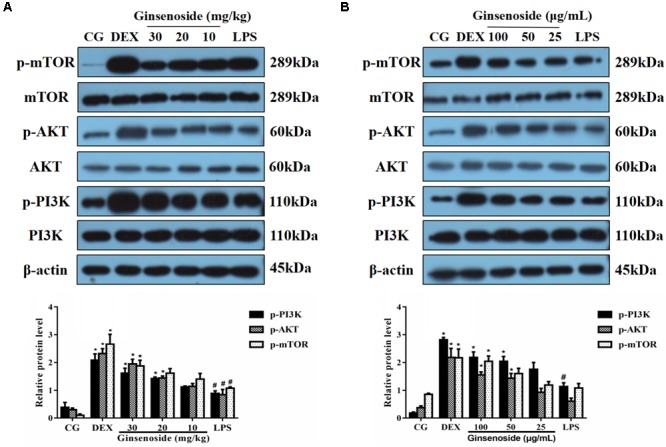
Effects of ginsenoside Rg3 on LPS-induced activation of the PI3K/AKT/mTOR pathway in WT groups. **(A)** The levels of PI3K, AKT and mTOR proteins in lung tissues were measured by western blotting. **(B)** The levels of PI3K, AKT and mTOR proteins in RAW264.7 cells were measured by western blotting. β-actin was used as a control. CG is the control group. LPS is the LPS-stimulated group. DEX is the dexamethasone group. The data are presented as the mean ± SEM of three independent experiments. ANOVA, *p* < 0.01, *post hoc* #*p* < 0.05 vs. CG. *^∗^p* < 0.05 vs. LPS group.

The activation of the PI3K/AKT/mTOR pathway induced by LPS was assessed to investigate the specific anti-inflammatory mechanism of ginsenoside Rg3. These results indicated that LPS increased the quantity of the expression of the phosphorylation of PI3K, AKT and mTOR in lung tissues, consistent with previous studies (Ohtani et al., 2008; Mendes Sdos et al., 2009). However, in ginsenoside Rg3 and DEX treatment groups, the phosphorylation levels of these proteins showed an overall increased trend when compared with the LPS-treatment group (**Figure 3A**). To further confirm the results presented in lung tissues, the same studies were performed on RAW264.7 cells, and similar results were observed, with only high or medium doses of ginsenosides Rg3 treatment significantly affecting the level of phosphorylation of these proteins (**Figure 3B**).

### Effects of Ginsenoside Rg3 on MerTK Expression in WT Groups

**FIGURE 4:**
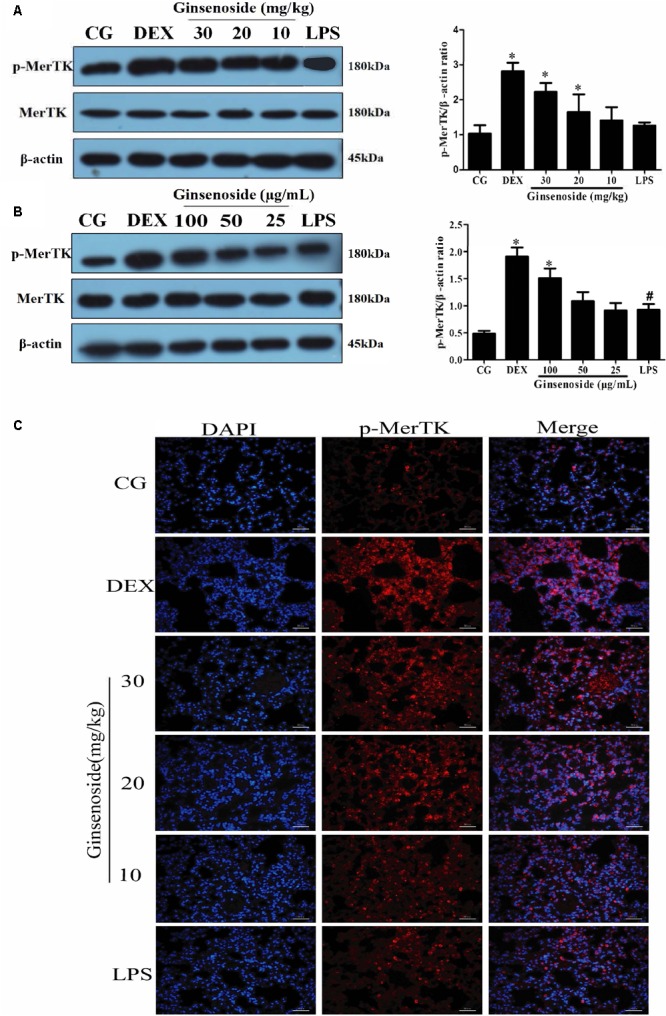
Effects of ginsenoside Rg3 on the expression of MerTK in WT groups. **(A)** The expression of MerTK protein in lung tissues was measured by western blotting. **(B)** The expression of MerTK protein in RAW264.7 cells was measured by western blotting. **(C)** The expression of MerTK protein in lung tissues was measured by immunofluorescence. p-MerTK protein was labeled with a red fluorophore, and the cell nucleus was labeled with a blue fluorophore. CG is the control group. LPS is the LPS-stimulated group. DEX is the dexamethasone group. Ginsenoside (10, 20, and 30) represent ginsenoside Rg3 (10, 20, and 30mg/kg) + LPS in animals and Ginsenoside (25, 50, and 100) represent ginsenoside Rg3 (25, 50, and 100 μg/mL) + LPS in cells. The data are presented as the mean ± SEM of three independent experiments. ANOVA, *p* < 0.05, *post hoc* ^#^*p* < 0.05 vs. CG. *^∗^p* < 0.05 vs. LPS group.

To further explore whether MerTK could play a vital role in ginsenoside Rg3-dependent immunomodulatory effects, we detected the expression levels of the phosphorylation of MerTK in lung tissues of WT mice and RAW264.7 cells. After LPS stimulation, the expression levels of p-MerTK were increased in both lung tissues and RAW264.7 cells. In contrast, p-MerTK protein significantly increased after ginsenoside Rg3 and DEX treatment (**Figures 4A,B**). Similarly, immunofluorescence was applied to investigate the expression levels of p-MerTK in lung tissues. Compared with the control group, the expression levels of p-MerTK in the LPS-induced group exhibited a significant decrease. Meanwhile, the expression levels of p-MerTK obviously increased in ginsenoside Rg3 and DEX treatment groups (**Figure 4C**).

### Ginsenoside Rg3 Does Not Have an Anti-inflammatory Effect in MerTK^-/-^ Mice After ALI

**FIGURE 5:**
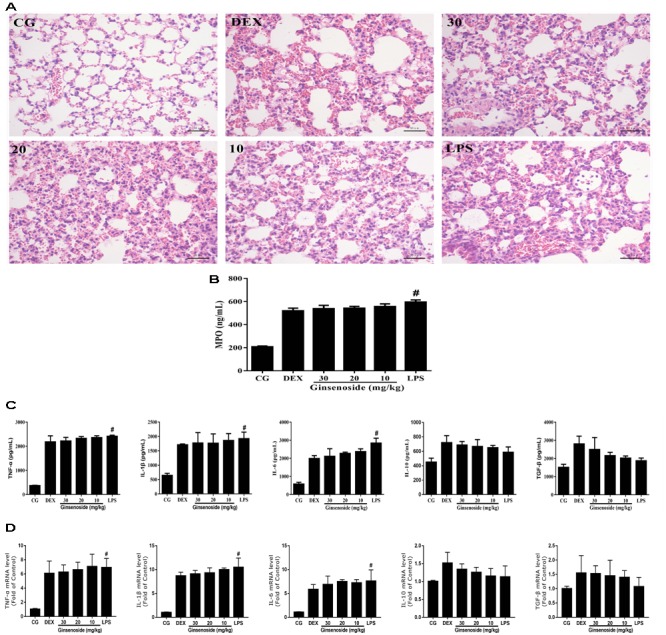
Anti-inflammatory effects of ginsenoside Rg3 in MerTK^-/-^ mice. **(A)** Histopathological analysis of lung tissues in MerTK^-/-^ mice. **(B)** The production of MPO in lung tissues in MerTK^-/-^ mice. **(C)** The protein expression levels of TNF-α, IL-1β, IL-6, IL-10, and TGF-β in lung tissues of MerTK^-/-^ mice were measured by ELISA. **(D)** The mRNA expression levels of TNF-α, IL-1β, IL-6, IL-10, and TGF-β in lung tissues of MerTK^-/-^ mice were measured by qPCR. β-actin was used as a control. CG is the control group. LPS is the LPS-stimulated group. DEX is the dexamethasone group. Ginsenoside (10, 20, and 30) represent ginsenoside Rg3 (10, 20, and 30mg/kg) + LPS in animals. The data are presented as the mean ± SEM of three independent experiments. ANOVA, *p* < 0.01, *post hoc* ^#^*p* < 0.05 vs. CG.

To further confirm whether the anti-inflammatory effects of ginsenoside Rg3 were mediated through MerTK, MerTK^-/-^ mice were used in the present study. In MerTK^-/-^ mice groups, it could be seen that pathological changes did not represent significant differences among LPS stimulated, ginsenoside Rg3 and DEX treatment groups, which still showed severe lung pathological damage (**Figure 5A**). MPO levels and the production of cytokines in lung tissues of MerTK^-/-^ mice also revealed no significant difference between the LPS treatment group and the medicine treatment groups (**Figures 5B–D**). These aforementioned results indicated that the anti-inflammatory effects of ginsenoside Rg3 are mediated by MerTK.

### PI3K/AKT/mTOR Pathway Activation Is Dependent on MerTK

**FIGURE 6:**
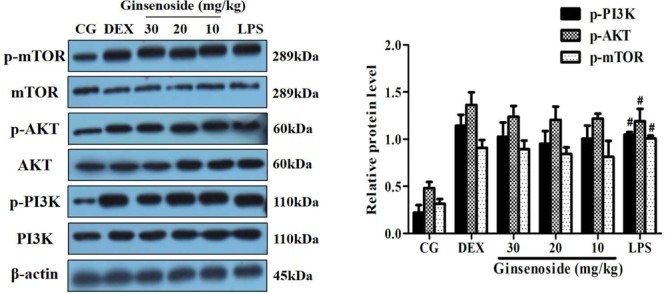
Effects of ginsenoside Rg3 on the LPS-induced activation of the PI3K/AKT/mTOR pathway in MerTK^-/-^ mice. The levels of PI3K, AKT and mTOR proteins in lung tissues were measured by western blotting. CG is the control group. LPS is the LPS-stimulated group. DEX is the dexamethasone group. Ginsenoside (10, 20, and 30) represent ginsenoside Rg3 (10, 20, and 30 mg/kg) + LPS in animals. The data are presented as the mean ± SEM of three independent experiments. ANOVA, *p* < 0.01, *post hoc* ^#^*p* < 0.05 vs. CG.

To demonstrate whether the effects of ginsenoside Rg3 attenuating LPS-induced lung injury were mediated by the MerTK-dependent PI3K/AKT/mTOR pathway, MerTK^-/-^ mice were used to assess the protein expression levels of the phosphorylation of PI3K, AKT and mTOR. As is shown in **Figure 6**, the expression levels of these phosphorylated proteins showed no significant differences among the LPS injection group, ginsenoside Rg3 treatment group and DEX treatment group, while the phosphorylation of these proteins remarkably increased after LPS induction compared with the control group. These results further proved that ginsenoside Rg3 promotes LPS-induced activation of the PI3K/AKT/mTOR pathway, which is dependent on MerTK.

## Discussion

ALI remains a major problem in clinical disease (Zimmermann et al., 2017), which affects the health of most humans (Rubenfeld et al., 2005). Ginsenoside Rg3, a traditional herbal medicine, is mainly involved in curing lung cancer (Kim et al., 2014). Some recent research has verified that ginsenoside Rg3 is supposed to have anti-tumor, anti-inflammatory and anti-fatigue activities (Bi et al., 2017; Guan and Xu, 2017). However, research with regards to the specific mechanism by which ginsenoside Rg3 takes effect on the anti-inflammatory process is rarely reported. In the present study, we established a mouse model of ALI to explore the possible mechanism of ginsenoside Rg3 in the inflammatory response induced by LPS.

In the present study, we demonstrated that ginsenoside Rg3 exerted an anti-inflammatory function, consistent with a previous study (Hien et al., 2010). We found that ginsenoside Rg3 treatment could relieve inflammation despite serious lung pathological damage induced by LPS according to the histological results. MPO is mainly synthesized and expressed by neutrophils (Sabharwal et al., 1995), and it is widely considered to be the main symbol of neutrophil activation and recruitment in inflammatory reaction (Klebanoff, 2005; Jiang et al., 2017). Some studies have suggested that the levels of MPO could be regarded as an indicator of increased risk for local and systemic inflammation (Nizam et al., 2014; Wang et al., 2014). As it is shown by the results, the production of MPO was obviously increased after LPS stimulation, which was consistent with previous studies (Menghini et al., 2016; Locatelli et al., 2017). Ginsenoside Rg3 treatment could dose-dependently decrease the production of MPO in lung tissues, indicating that ginsenoside Rg3 could interpose neutrophil activation and recruitment after LPS challenge.

Neutrophils and macrophages, which play a major role in the inflammatory response in LPS-induced ALI, are the primary source of diversified inflammatory mediators such as pro-inflammatory cytokines TNF-α, IL-1β and IL-6 as well as anti-inflammatory cytokines IL-10 and TGF-β (Goodman et al., 2003; Fligiel et al., 2006). In the study presented here, we found that ginsenoside Rg3 significantly inhibited the increases of the pro-inflammatory cytokines TNF-α, IL-1β and IL-6 as well as prevented the decreases of anti-inflammatory cytokines IL-10 and TGF-β after LPS stimulation. The process by which macrophage activation induced different subtypes to respond to changes in the environment is generally called polarization, and the phenotype of polarization can be divided into M1 and M2 (Xie et al., 2016). The polarization of M2 macrophages appears to promote the release of anti-inflammatory and immunosuppressive agents as well as motivate the repair and healing of damaged tissue (Arnold et al., 2007; Kang et al., 2008). Intriguingly, ginsenoside Rg3 treatment also led to an increase in LPS-induced M2 polarization. Taken together, ginsenoside Rg3 exerted anti-inflammatory action through decreasing the production of pro-inflammatory cytokines, increasing the expression levels of anti-inflammatory mediators and promoting M2 polarization.

The PI3K/AKT/mTOR signaling pathway is involved in the antitumor effects of ginsenoside Rg3 in lung cancer cells (Xie et al., 2017). Ginsenoside Rg3 inhibits angiogenesis in a rat model of endometriosis through the VEGFR-2-mediated PI3K/Akt/mTOR signaling pathway (Cao et al., 2017). Recent studies also have revealed that activation of the PI3K/AKT pathway exhibits a protective effect in a mouse model of ALI. However, there is no report on whether ginsenoside Rg3 can attenuate LPS-induced ALI via the PI3K/Akt/mTOR signaling pathway. In our study, ginsenoside Rg3 treatment increased the expression of phosphorylated PI3K, AKT and mTOR in LPS-challenged mice. PI3K/Akt has been shown to suppress the activation of NF-κB, which eventually prevents the occurrence of inflammation. NF-κB is a multifunctional nuclear transcriptional factor, which can regulate a series of cell survival and apoptosis related gene products (Chen et al., 1999). The synthese of pro-inflammatory cytokines TNF-α, IL-1β, IL-6 and IL-8 are mediated by NF-κB, and these pro-inflammatory cytokines are upregulated by NF-κB transcriptional activity (Tak and Firestein, 2001; Koh et al., 2017). It has been well established that ginsenoside Rg3 can mitigate inflammation via the NF-κB pathway (Lee et al., 2016). Interestingly, Ginsenoside Rg3 has also been reported to ameliorate ALI through inactivating the NF-κB signaling pathway (Cheng and Li, 2016). A previous study has demonstrated that mTOR acts downstream of the PI3K/AKT pathway and restrains the effects of NF-κB in LPS-induced RAW264.7 macrophages (Mendes Sdos et al., 2009). Furthermore, the PI3K/AKT/mTOR pathway positively regulates the production of IL-10 (Ohtani et al., 2008), which is thought to be a pleiotropy anti-inflammatory mediator (Zigmond et al., 2014). Thus, the results may be explained by the fact that the PI3K/AKT/mTOR pathway is activated by ginsenoside Rg3 and then downregulates NF-κB to restrain the transcription of pro-inflammatory mediators as well as upregulates the production of anti-inflammatory cytokines.

The recent studies have indicated that MerTK has a negative regulatory effect on the LPS-induced the inflammatory response (Lee et al., 2012a). It has been reported that 88.2% of MerTK^-/-^ mice die of releasing excess TNF-α due to LPS stimulation (Camenisch et al., 1999), and RAW264.7 cells treated with the anti-MerTK antibody enhancing the production of inflammatory cytokines (Lee et al., 2012a). More importantly, MerTK plays a critical role in the activation of PI3K/Akt. There is growing evidence that MerTK mediates the activation of PI3K/Akt pathway in macrophages, and then results in the blockade of NF-κB (Eken et al., 2010). Stimulation of Mer pathway by Gas6 in U937 cells also gives rise to an increased phosphorylation of Akt (Alciato et al., 2010). In addition, activation of MerTK negatively regulates TLR2-mediated immune response via PI3K/Akt pathway and SOCS3 protein (Zhang et al., 2016). To further explore the specific molecular mechanism through which ginsenoside Rg3 exerts its anti-inflammatory effect, we measured the MerTK-dependent PI3K/AKT/mTOR signaling pathway. In our study, the expression levels of phosphorylated MerTK significantly increased after ginsenoside Rg3 treatment in both the immunofluorescence and western blot results. Furthermore, the results in MerTK^-/-^ mice showed the same pathological damage, the production of MPO and inflammatory cytokines, whether for the LPS treatment group or ginsenoside Rg3 treatment groups, and there is no significant difference with regards to the protein expression levels of phosphorylated PI3K, AKT and mTOR between the LPS treatment group and ginsenoside Rg3 treatment groups. These results potently suggest that the anti-inflammatory effects of ginsenoside Rg3 were mediated via the MerTK-dependent PI3K/AKT/mTOR signaling pathway.

## Conclusion

The present study demonstrated that ginsenoside Rg3 could attenuate LPS-induced ALI, and the possible molecular mechanism of the protective effects of ginsenoside Rg3 in ALI is probably because of the regulatory effect of MerTK-mediated activation of its downstream PI3K/AKT/mTOR pathway. Our findings may provide further understanding with regards to the anti-inflammatory effects of ginsenoside Rg3, which may be a potentially powerful drug to cure LPS-induced ALI.

## Author Contributions

RF conceived and designed the study. JY drafted the manuscript. JY, SL, LW, FD, XZ, QS, and JZ performed the experiments and analyzed the data. All authors read and approved the final manuscript.

## Conflict of Interest Statement

The authors declare that the research was conducted in the absence of any commercial or financial relationships that could be construed as a potential conflict of interest.
